# Supplementary material for: Cobalt protoporphyrin IX increases endogenous G‐CSF and mobilizes HSC and granulocytes to the blood
Source: EMBO Mol Med. 2019 Nov 11;11(12):e09571. doi: 10.15252/emmm.201809571 (PMC6895613; doi:10.15252/emmm.201809571)
Supplement: Supplementary file 1 — Appendix [file EMMM-11-e09571-s001.pdf]

Table of contents:

Figure S1. Plasma concentrations of selected growth factors in plasma of C3H mice treated with CoPP and SnPP measured by Luminex assay.

Figure S2. The percentage of cells isolated from C57BL/6xFVB mice treated with G-CSF or CoPP that are producing reactive oxygen species after incubation with indicated stimuli.

Figure S3. KLS cells in the blood and bone marrow.

Figure S4. Complete blood cell count in recipient mice 4 weeks after mobilized blood transplantation.

Figure S5. G-CSF is increased in the spleen after CoPP treatment.

Figure S6. Monocytes percentage in PB of $Nrf2^{+/+}$ and $Nrf2^{-/-}$ mice.

Figure S7. Quantum yield of singlet oxygen formation measured by the laser flash photolysis.

Figure S8. Inflammation and toxicity markers measured in plasma of C57BL6xFVB mice injected with G-CSF, CoPP or solvent controls.

Figure S9. Example analysis of GFP chimerism in peripheral blood of mice after mobilized blood transplantation.

Figure S10. Example of gating strategy of granulocytes in the blood.

Figure S11. Example of gating strategy of $CD11b^{+}F4/80^{+}MHCl^{ow}Ly6G^{+}$ macrophages in the bone marrow.

Figure S12. Example gating of HSPC populations in PB.

Figure S13. Example analysis of main leukocyte populations in blood.

Figure S14. Gating strategy for Phagoburst assay analysis.

Table S1. Antibodies and reagents used for flow cytometry.

Table S2. Exact p values and statistical tests used.

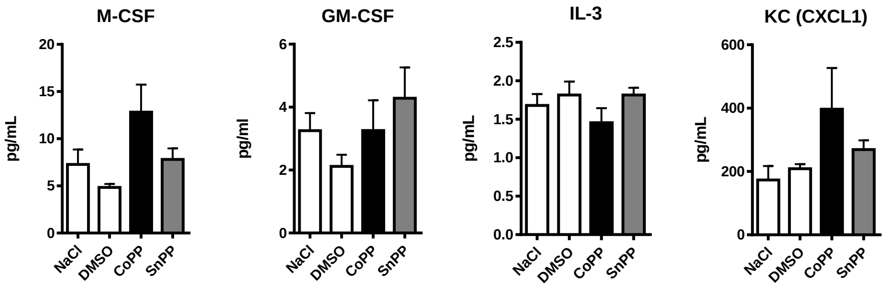

Appendix Figure S1. Plasma concentrations of selected growth factors in plasma of C3H mice treated with CoPP and SnPP measured by Luminex assay.

C3H mice were injected with CoPP, SnPP or solvent controls (NaCl, DMSO) each second day for 5 days. Samples were collected 24 hours after the last injection. Results are shown mean + SEM, one-way Anova with Bonferroni post-test, n = 5 mice per group.

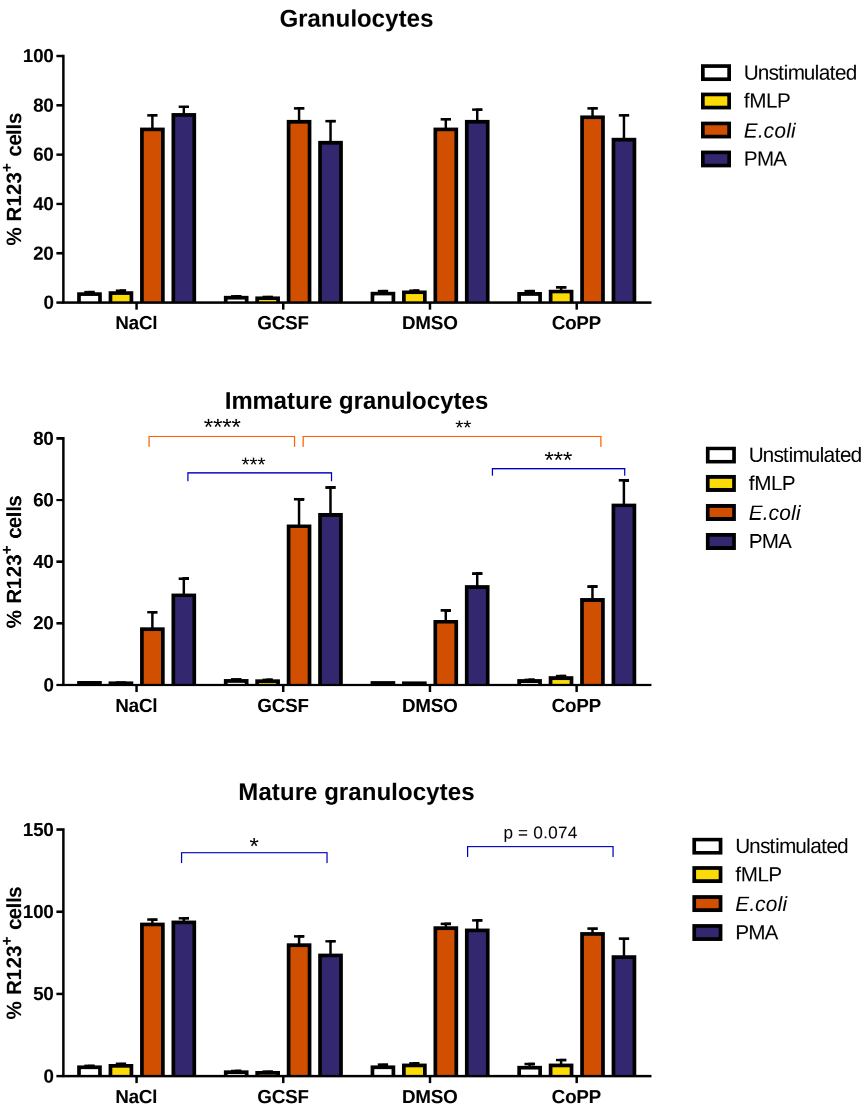

Appendix Figure S2. The percentage of cells isolated from C57BL/6xFVB mice treated with G-CSF or CoPP that are producing reactive oxygen species after incubation with indicated stimuli (mean + SEM, two-way Anova with Bonferroni post-test, n = 6 mice per group). Total granulocytes from mice treated with G-CSF and CoPP show similar ROS production as granulocytes from control mice upon stimulation with *E.Coli* or PMA. Granulocytes with immature phenotype mobilized with CoPP and G-CSF and stimulated with PMA showed higher ROS production than non-mobilized immature phenotype granulocytes, however only G-CSF mobilzed immature granulocytes showed higher ROS production upon *E.Coli* stimulation. Among granulocytes with mature phenotype, after G-CSF mobilization the ROS production decresed upon PMA stimulation, while only trend to decreased ROS production was observed in CoPP-mobilized mature phenotype. The ROS production upon *E.Coli* stimualtion in mature granulocytes did not differ between mobilized and nonmobilized group.

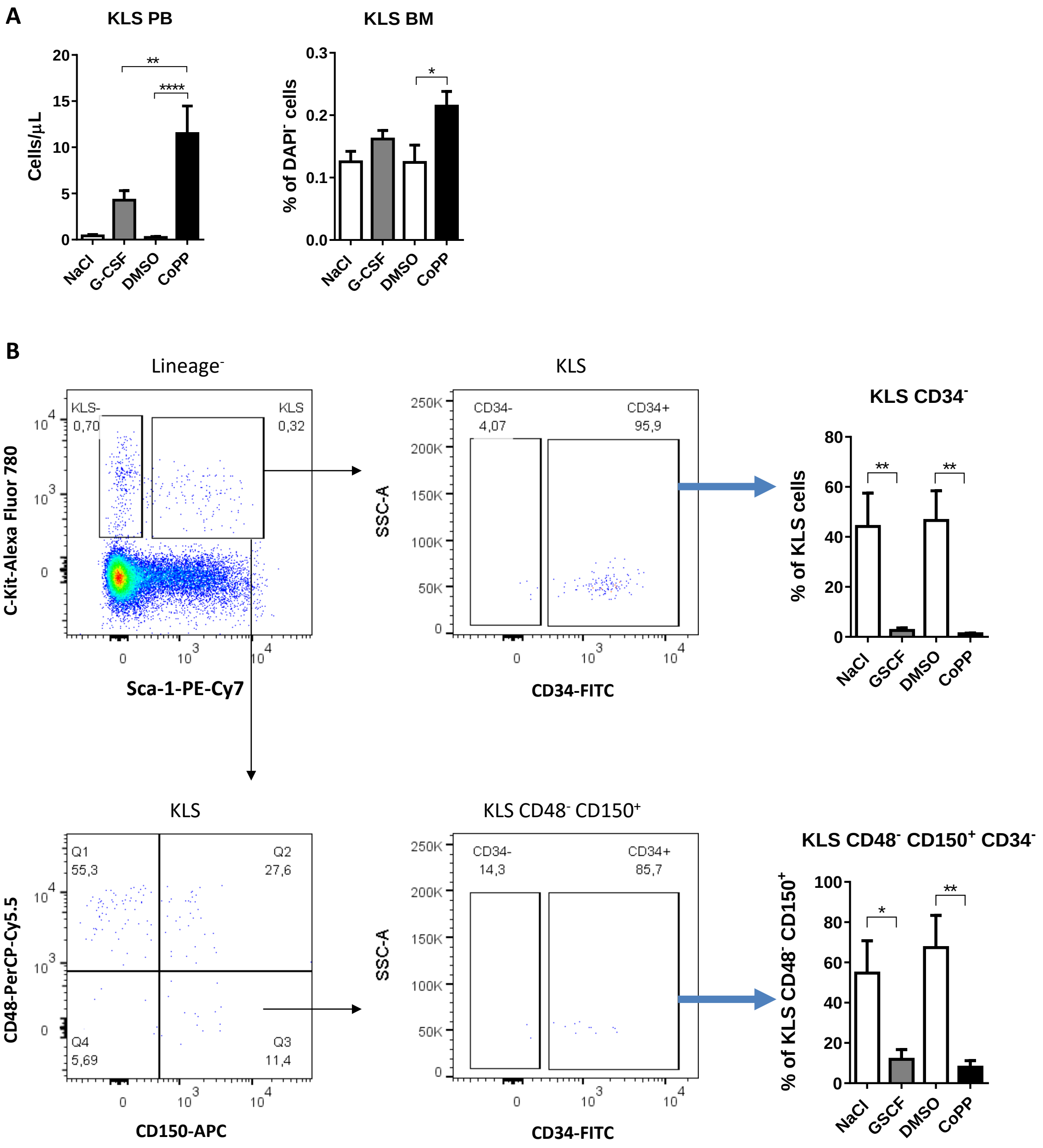

Appendix Figure S3. KLS cells in the blood and bone marrow. (A) Number of KLS (c-Kit$^+$Lin$^-$Sca-1$^+$) cells in blood and percentage of KLS cells in the bone marrow **(B)** Percentage of CD34$^-$ cells among KLS (c-Kit$^+$Lin$^-$Sca-1$^+$) and KLS CD48$^-$CD150$^+$ populations in blood of mice mobilized with G-CSF or CoPP Results are shown as mean + individual values plotted, one-way Anova with Bonferroni post-test, n = 7 mice per group.

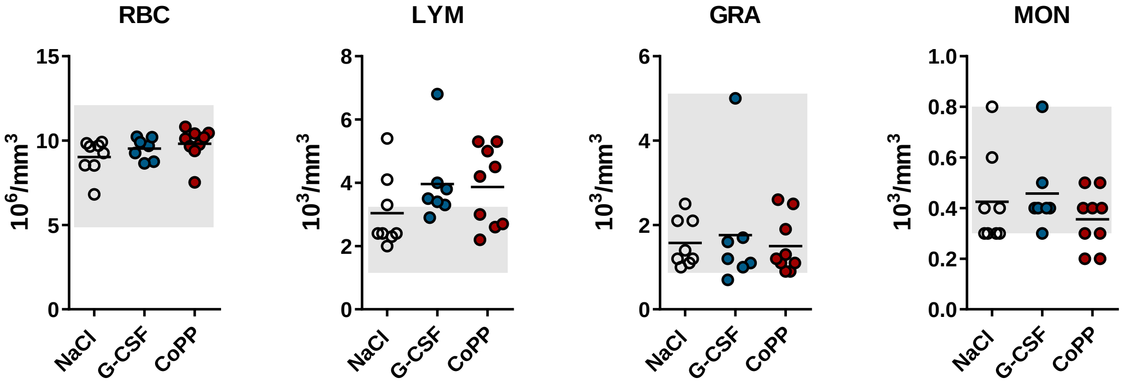

Appendix Figure S4. Complete blood cell count in recipient mice 4 weeks after mobilized blood transplantation. Donor GFP+ mice were treated with NaCl, G-CSF or CoPP daily. At the 5th day of treatment we transplanted $5x10^6$ of isolated PBMC to the lethally irradiated GFP- recipient mice, together with 10^5 GFP- BM-derived competitor cells. Four weeks after transplantation lymphocytes, granulocytes, monocytes,and RBC values reached the physiological range, without differences between the groups (mean + individual values, one-way Anova with Bonferroni post-test, NaCl: n = 8, G-CSF: n = 7, CoPP: n = 9 mice per group).

RBC – number of red blood cells
LYM – number of lymphocytes
GRA – number of granulocytes
MON – number of monocytes.

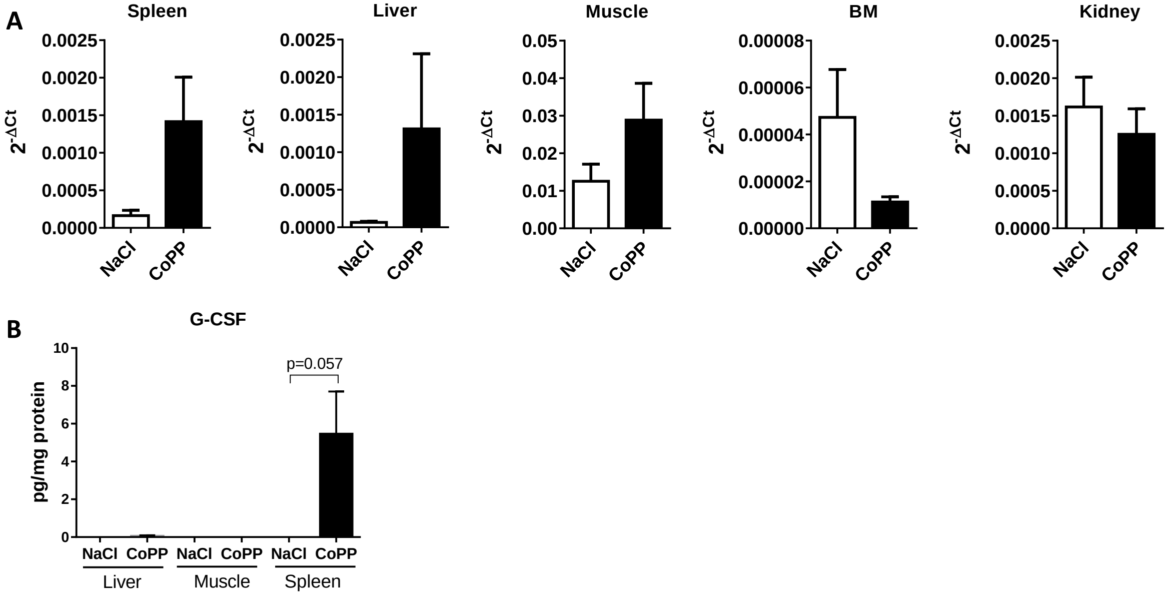

Appendix Figure S5. G-CSF is increased in the spleen after CoPP treatment
mRNA for G-CSF was detectable in the spleen, liver and muscle lysate **(A)** whereas G-CSF protein was incresed only in the spleen lysate **(B)** after CoPP treatment (mean + SEM, unpaired two-tailed T-test, NaCl: n = 5, CoPP: n = 6 mice per group).

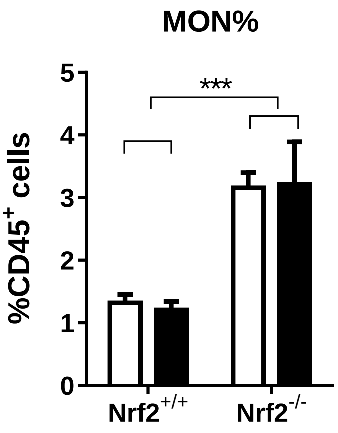

Appendix Figure S6. Monocytes percentage in PB of Nrf2$^{+/+}$ and Nrf2$^{-/-}$ mice. Nrf2-deficient and control C57BL/6 mice were injected with CoPP everyday for 5 days and sacrificed 6 hours after last injection (mean + SEM, two-way Anova with Bonferroni post-test, DMSO: n = 4, CoPP: n = 5 mice per group).

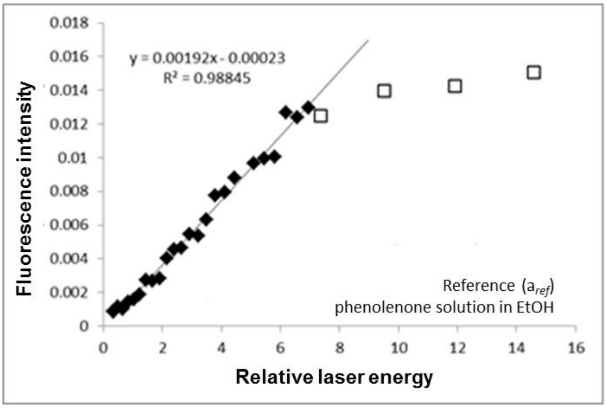

$$\Phi_\Delta = \Phi_{\Delta\,ref} \cdot \frac{a}{a_{ref}}$$

Φ_Δ SnPP	Φ_Δ CoPP
0.89	0

Appendix Figure S7. Quantum yield of singlet oxygen formation measured by the laser flash photolysis.

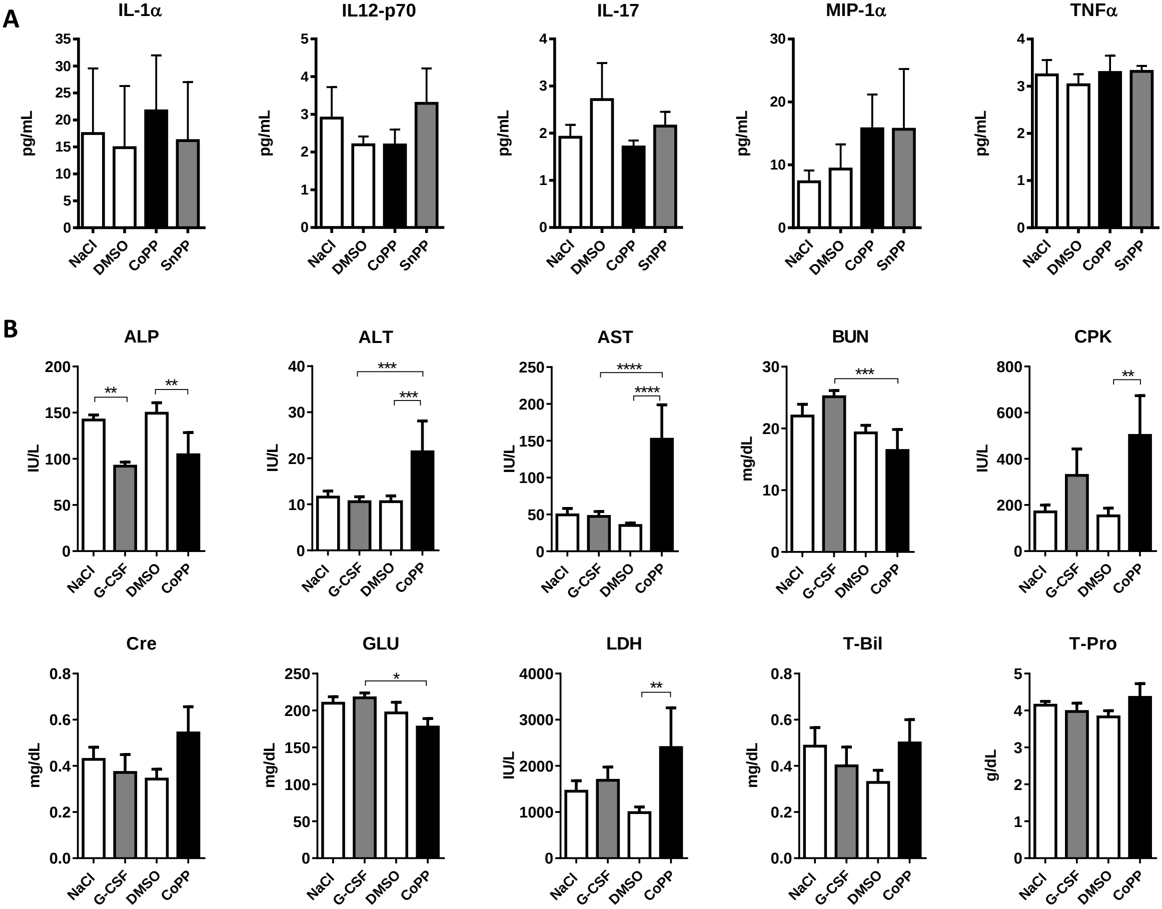

Appendix Figure S8. Inflammation and toxicity markers measured in plasma of C57BL6xFVB mice injected with G-CSF, CoPP or solvent controls (NaCl, DMSO) every day for 5 days. Samples were collected 6 hours after the last injection and frozen at -80°C (mean + SEM, one-way Anova with Bonferroni post-test, 7 mice per group). **(A)** Concentrations of proinflammatory cytokines measured by Luminex assay.
(B) Liver and kidney muscle toxicity markers measured using SpotChem EZ Chemistry Analyzer (ALP - alkaline phosphatase, ALT - alanine transaminase, Cre - creatinine, GLU- glucose, T-Pro - total protein, BUN - blood urea nitrogen, ALT - aspartate transaminase, CPK - creatine kinase, LDH - lactate dehydrogenase, T-Bil - total bilirubin). Amounts of gamma-GT were undetectable.

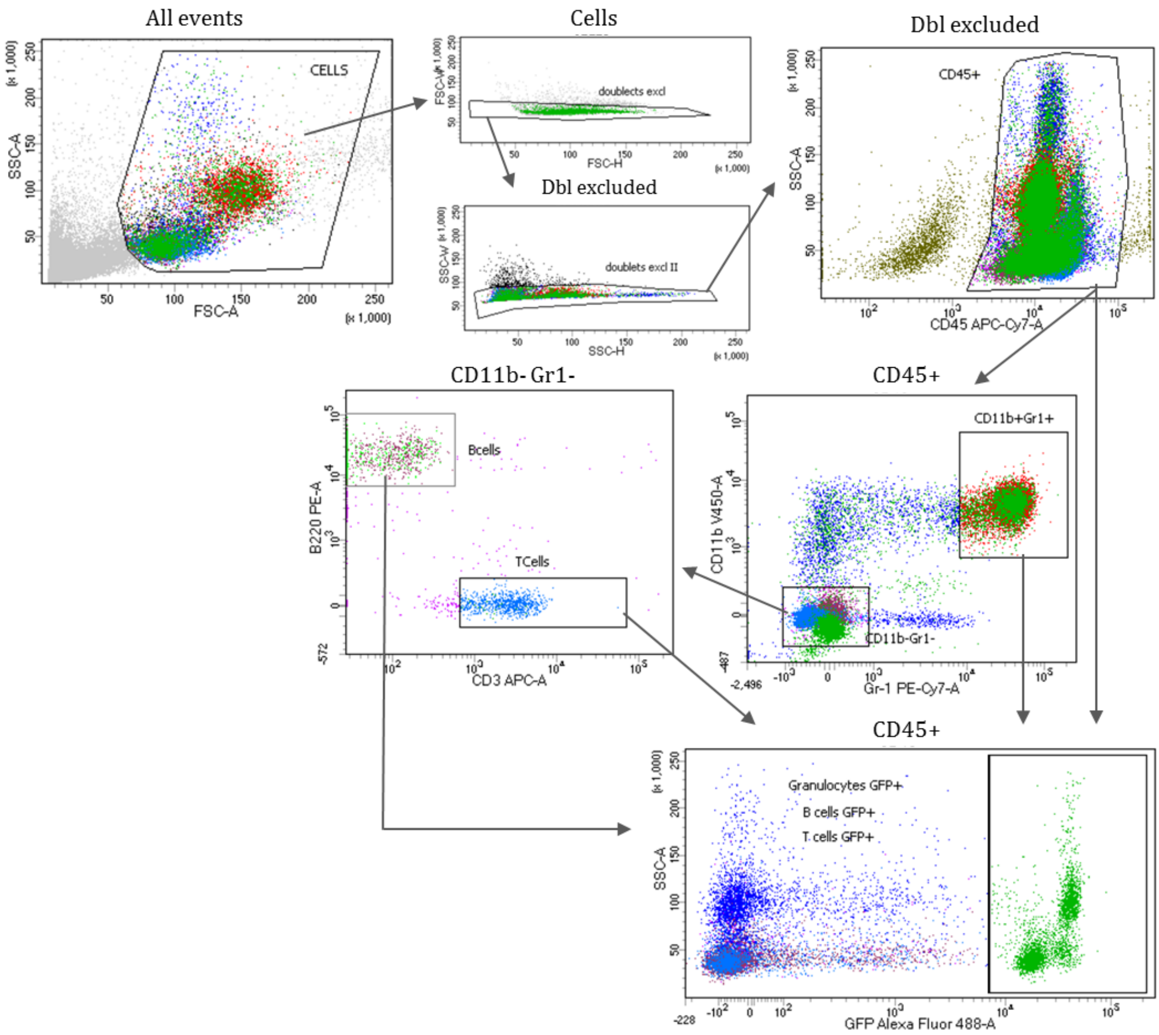

Appendix Figure S9. Example analysis of GFP chimerism in peripheral blood of mice after mobilized blood transplantation.

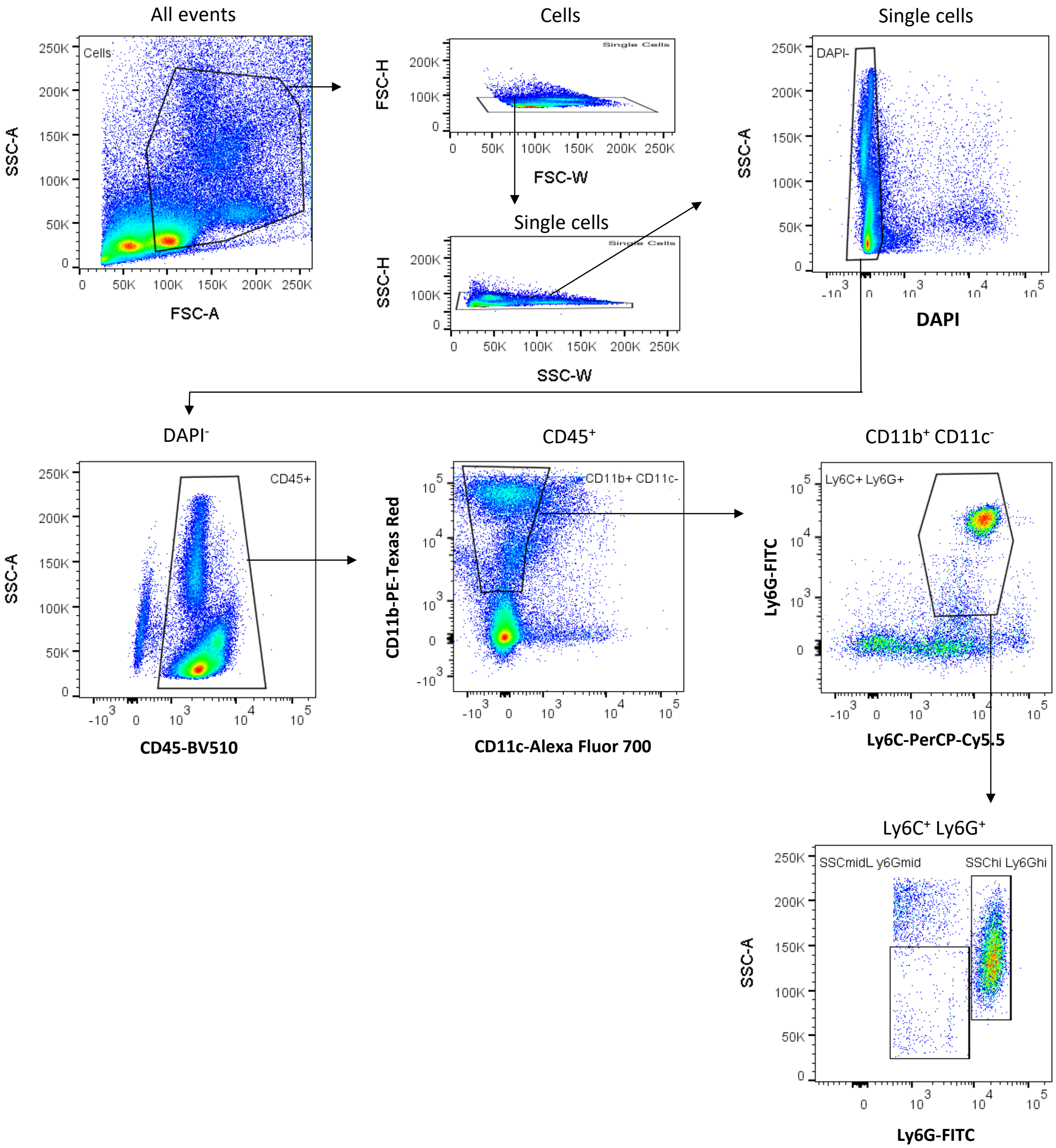

Appendix Figure S10. Example of gating strategy of granulocytes in the blood.

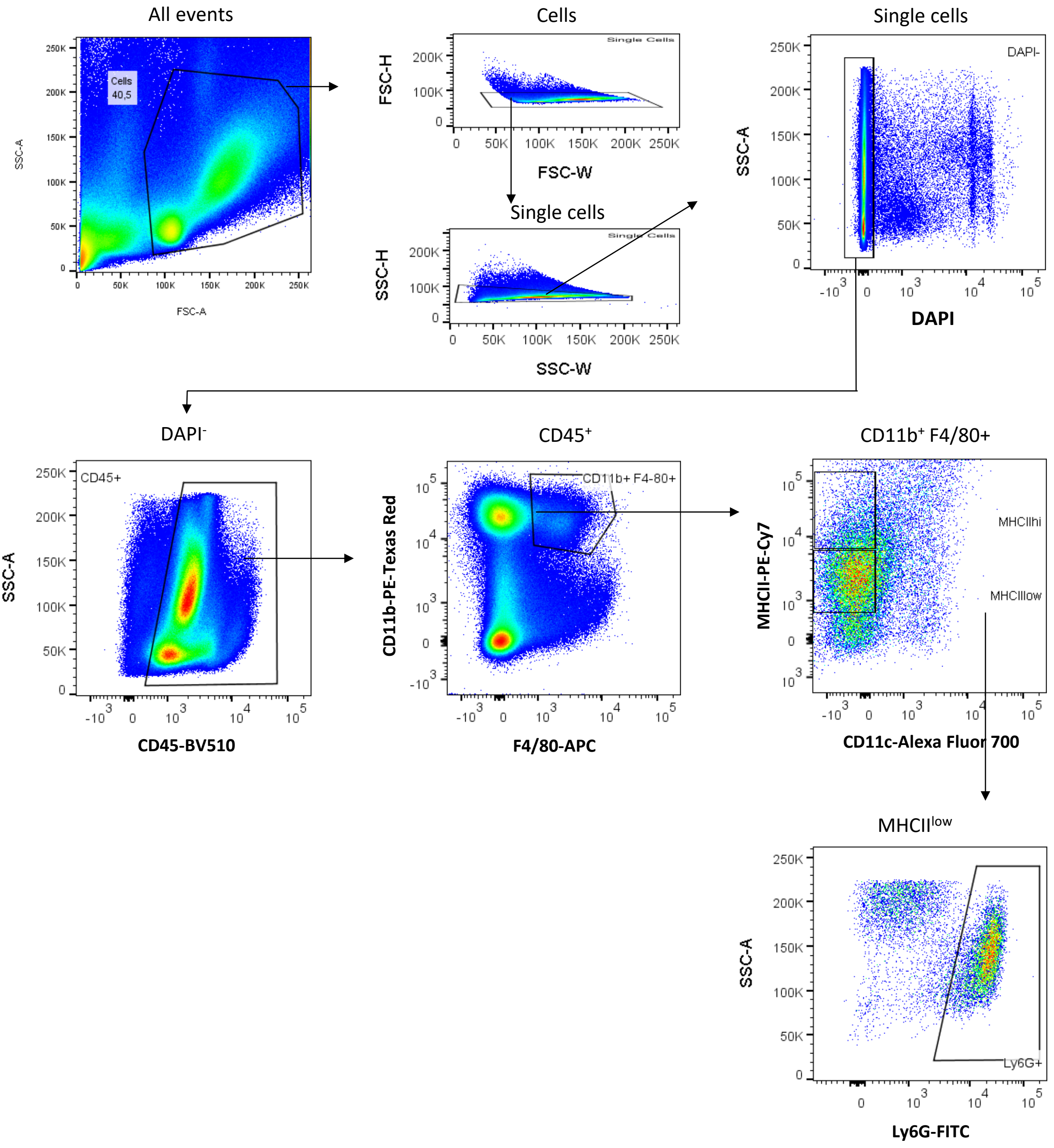

Appendix Figure S11. Example of gating strategy of CD11b$^+$F4/80$^+$MHCl^{ow}Ly6G$^+$ macrophages in the bone marrow.

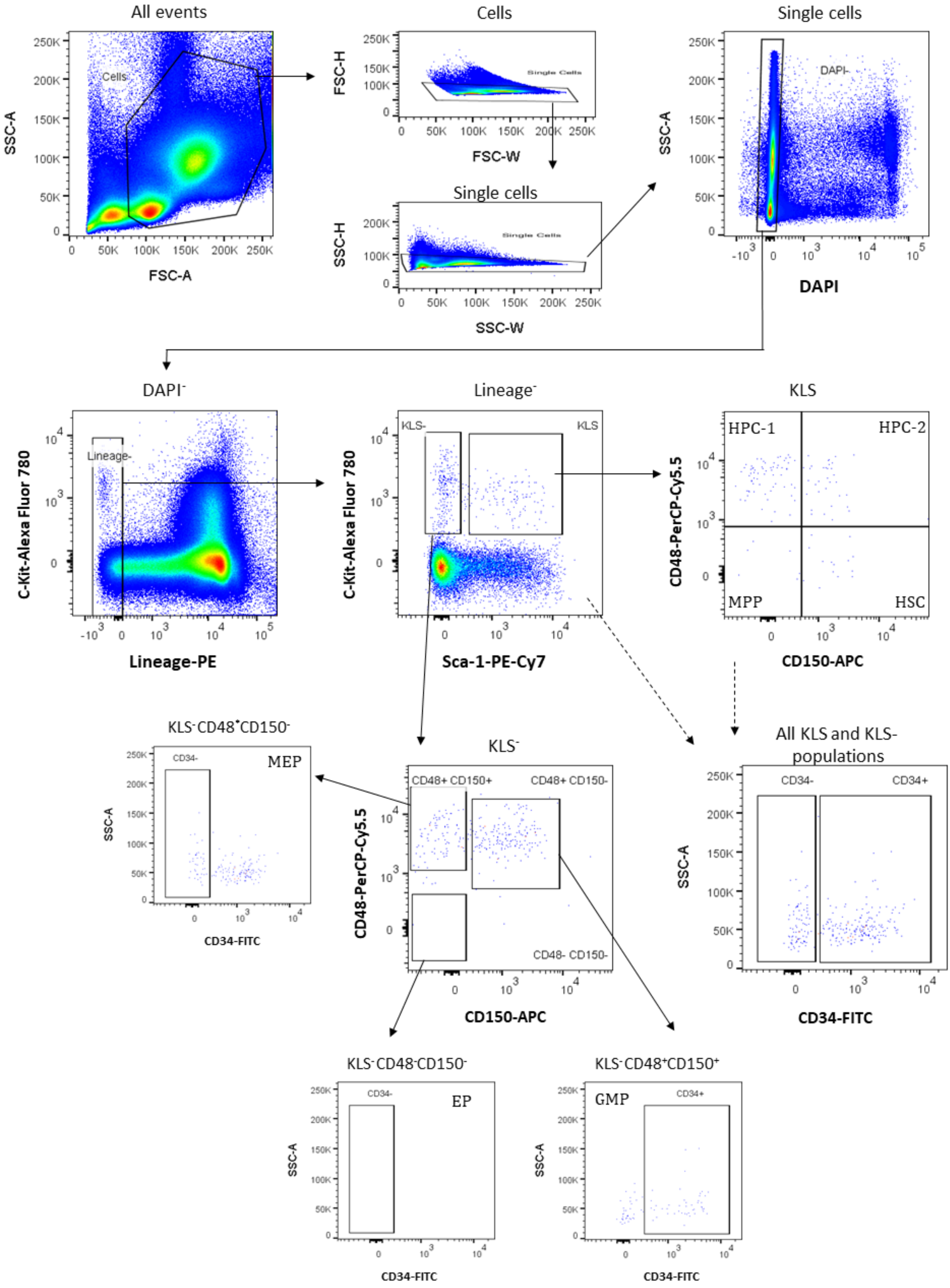

Appendix Figure S12. Example gating of HSPC populations in PB.

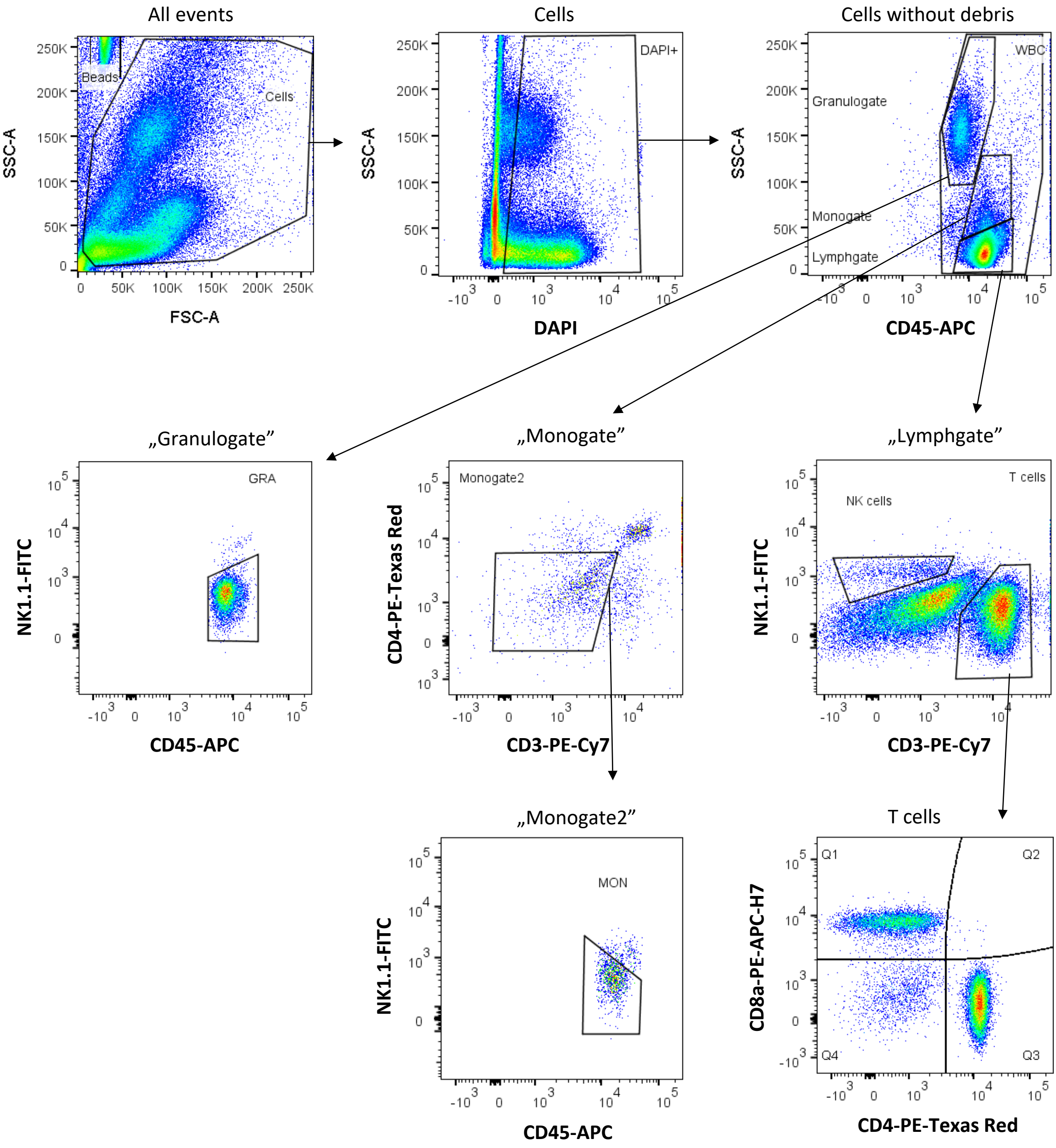

Appendix Figure S13. Example analysis of main leukocyte populations in blood. BD Trucounts were used to calculate total number of gated cells per µl of PB. PB was fixed with BD FACS Lysing Solution.

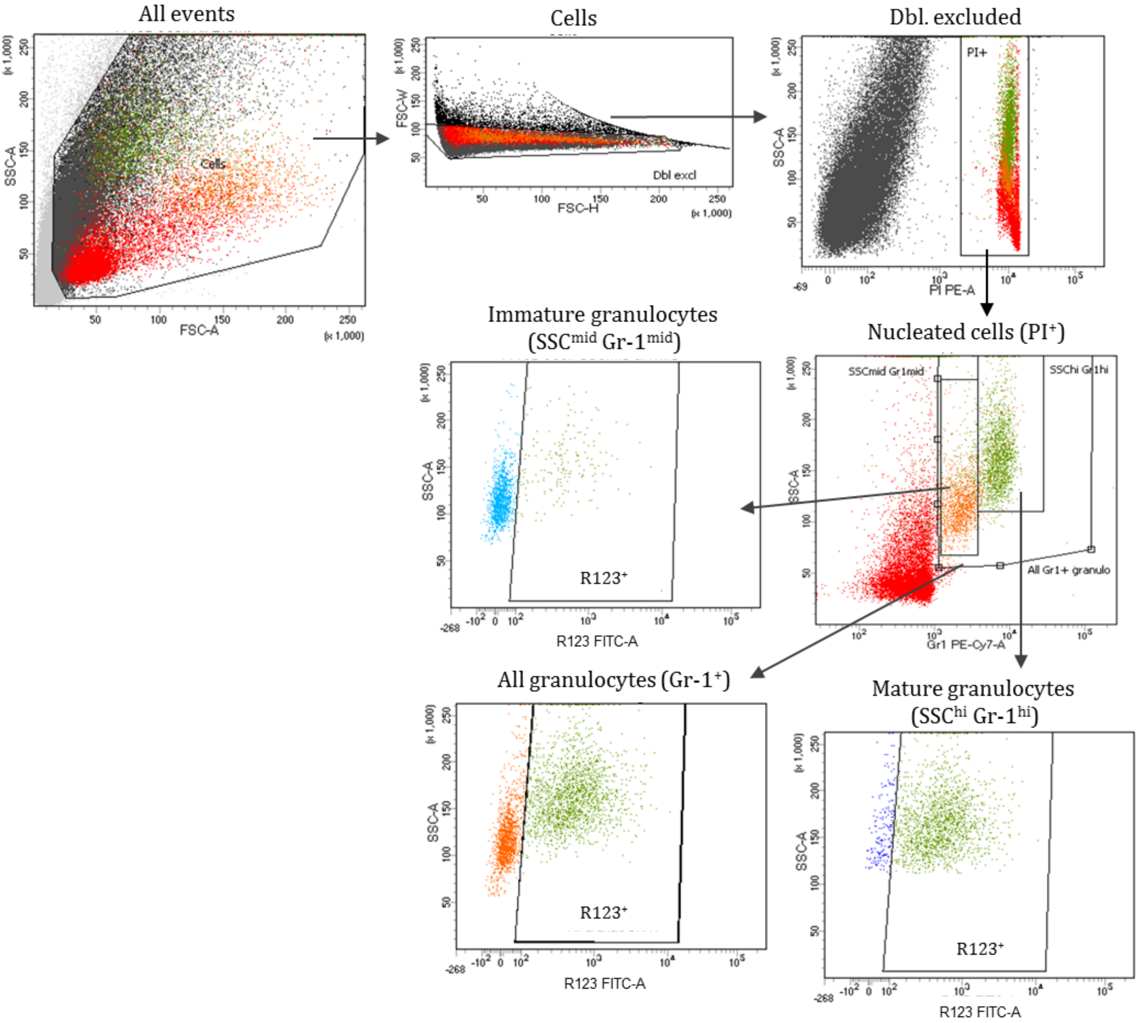

Appendix Figure S14. Gating strategy for Phagoburst assay analysis.

Antigen	Dye	Clone	Company	Dilution
CD3e	PE-Cy7	145-2C11	BD Biosciences	1:50
CD3e	APC	17A2	BD Biosciences	1:50
CD4	PE-CF594	RM4-5	BD Biosciences	1:50
CD8a	APC-H7	53-6.7	BD Biosciences	1:50
CD8a	APC-Cy7	53-6.7	BD Biosciences	1:50
CD11b	PE	M1/70	BD Biosciences	1:50
CD11b	V450	M1/70	BD Biosciences	1:50
CD11b	PE-CF594	M1/70	BD Biosciences	1:50
CD11c	AlexaFluor700	N418	eBioscience	1:50
CD14	APC-Cy7	Sa14-2	Biolegend	1:50
CD25	PE	PC61	BD Biosciences	1:50
CD34	AlexaFluor700	RAM34	BD Biosciences	1:50
CD34	FITC	RAM34	BD Biosciences	1:50
CD43	FITC	S7	BD Biosciences	1:50
CD43	PE	S7	BD Biosciences	1:50
CD45	Pacific Orange	30F-10	Invitrogen	1:50
CD45	FITC	30F-11	BD Biosciences	1:50
CD45	APC	30F-11	BD Biosciences	1:50
CD45	APC-Cy7	30F-11	BD Biosciences	1:50
CD48	PerCP-Cy5.5	HM-48-1	Biolegend	1:50
CD150	APC	TC15-12F12.2	Biolegend	1:50
CD117 (c-Kit)	APC-eFluor780	2B8	eBioscience	1:50
CD202b (Tie-2)	PE	TEK4	eBioscience	1:50
I-A/I-E (MHC II)	PE-Cy7	M5/114.15.2	eBioscience	1:50
Ly-6A/E (Sca-1)	PE-Cy7	D7	BD Biosciences	1:50
Ly-6A/E (Sca-1)	PE-Cy5	D7	Biolegend	1:50
Ly6G/Ly6C (Gr-1)	PE-Cy7	RB6-8C5	BD Bioscience	1:50
Ly6C	PerCP-Cy5.5	HK1.4	eBioscience	1:50
Ly6C	PerCP-Cy5.5	AL.-21	BD Biosciences	1:50
Ly6G	FITC	1A8	BD Biosciences	1:50
Ly6G	BV605	1A8	BD Biosciences	1:50
Ly55 (NK1.1)	FITC	PK136	BD Biosciences	1:50
Ly71 (F4/80)	APC	BM8	eBioscience	1:50
Lineage staining				
CD11b	PE	M1/70	BD Biosciences	1:100
CD45R (B220)	PE	RA3-6B2	BD Biosciences	1:100
Ly6G/Ly6C (Gr-1)	PE	RB6-8C5	BD Biosciences	1:100
Ly76 (Ter119)	PE	TER-119	BD Biosciences	1:100
TCRβ	PE	H57-597	BD Biosciences	1:100
TCRγδ	PE	GL3	BD Biosciences	1:100
-	DAPI	-	Sigma	
-	Hoechst 33342	-	Sigma	

Appendix Table S1. Antibodies and reagents used for flow cytometry

Fig	Graph	Test	Comparison	Symbol	Exact p-value
1C	HO activity	one-way Anova with Bonferroni's multiple comparisons test	DMSO vs. CoPP	****	< 0.0001
			DMSO vs. SnPP	****	0.0009
			CoPP vs. SnPP	****	< 0.0001
1D	WBC	one-way Anova with Bonferroni's multiple comparisons test	DMSO vs. CoPP	****	< 0.0001
			CoPP vs. SnPP	****	< 0.0001
1D	%GRA	one-way Anova with Bonferroni's multiple comparisons test	DMSO vs. CoPP	****	< 0.0001
			CoPP vs. SnPP	****	< 0.0001
1D	%LYM	one-way Anova with Bonferroni's multiple comparisons test	DMSO vs. CoPP	****	< 0.0001
			CoPP vs. SnPP	****	< 0.0001
1F	G-CSF	one-way Anova with Bonferroni's multiple comparisons test	DMSO vs. CoPP	****	< 0.0001
			CoPP vs. SnPP	****	< 0.0001
1F	IL-6	one-way Anova with Bonferroni's multiple comparisons test	DMSO vs. CoPP		0.0734
			CoPP vs. SnPP		0.0751
1F	MCP-1	one-way Anova with Bonferroni's multiple comparisons test	DMSO vs. CoPP	**	0.0013
			CoPP vs. SnPP	**	0.0015
1F	IP-10	one-way Anova with Bonferroni's multiple comparisons test	DMSO vs. CoPP	****	< 0.0001
			CoPP vs. SnPP	****	< 0.0001
1F	IL-5	one-way Anova with Bonferroni's multiple comparisons test	DMSO vs. CoPP	*	0.0265
			CoPP vs. SnPP	*	0.0218
2A	CD45+ cells	one-way Anova with Bonferroni's multiple comparisons test	NaCl vs. G-CSF	****	< 0.0001
			G-CSF vs. CoPP	*	0.0346
			DMSO vs. CoPP	*	0.0155
2A	Granulocytes	one-way Anova with Bonferroni's multiple comparisons test	NaCl vs. G-CSF	****	< 0.0001
			G-CSF vs. CoPP	*	0.0179
			DMSO vs. CoPP	***	0.0004
2A	Monocytes	one-way Anova with Bonferroni's multiple comparisons test	NaCl vs. G-CSF	*	0.0115
			DMSO vs. CoPP	**	0.008
2A	Lymphocytes	one-way Anova with Bonferroni's multiple comparisons test	NaCl vs. G-CSF	*	0.0194
2A	T cells	one-way Anova with Bonferroni's multiple comparisons test	NaCl vs. G-CSF	*	0.0211
			G-CSF vs. CoPP	*	0.0161
2C	G-CSF	one-way Anova with Bonferroni's multiple comparisons test	DMSO vs. CoPP	**	0.0014
2C	IL-6	one-way Anova with Bonferroni's multiple comparisons test	G-CSF vs. CoPP	***	0.0005
			DMSO vs. CoPP	***	0.0006
2C	MCP-1	one-way Anova with Bonferroni's multiple comparisons test	DMSO vs. CoPP	**	0.007
2D	CD11b+ F4/80+ MHC II low Ly6G+	one-way Anova with Bonferroni's multiple comparisons test	NaCl vs. G-CSF	**	0.0015
			DMSO vs. CoPP	*	0.0115
2E	Spleen weight	one-way Anova with Bonferroni's multiple comparisons test	NaCl vs. G-CSF	***	0.0001
			DMSO vs. CoPP	**	0.0039
3B	Granulocytes	one-way Anova with Bonferroni's multiple comparisons test	NaCl vs. G-CSF	****	< 0.0001
			DMSO vs. CoPP	****	< 0.0001
3B	Ly6GmidSSCmid granulocytes	one-way Anova with Bonferroni's multiple comparisons test	NaCl vs. G-CSF	****	< 0.0001
			G-CSF vs. CoPP	****	< 0.0001
3B	Ly6GhiSSChi granulocytes	one-way Anova with Bonferroni's multiple comparisons test	NaCl vs. G-CSF	****	< 0.0001
			DMSO vs. CoPP	****	< 0.0001
3C	Ly6Clow Ly6G+	one-way Anova with Bonferroni's multiple comparisons test	NaCl vs. G-CSF	****	< 0.0001
			DMSO vs. CoPP	***	0.0007
			G-CSF vs. CoPP	**	0.0097

Appendix Table S2. Exact p values and statistical tests used.

Fig	Graph	Test	Comparison	Symbol	Exact p-value
3C	Ly6Clow SSCmed Ly6Gmed	one-way Anova with Bonferroni's multiple comparisons test	NaCl vs. G-CSF	****	< 0.0001
			DMSO vs. CoPP	****	< 0.0001
			G-CSF vs. CoPP	****	< 0.0001
3C	Ly6Clow SSChi Ly6Ghi	one-way Anova with Bonferroni's multiple comparisons test	NaCl vs. G-CSF	***	0.0004
3D	Oxidative burst	two-way Anova with Bonferroni's multiple comparisons test	E.coli: NaCl vs. G-CSF	*	0.0215
			E.coli: DMSO vs. CoPP	**	0.0072
			PMA: DMSO vs. CoPP		0.076
4B	HSC	one-way Anova with Bonferroni's multiple comparisons test	G-CSF vs. CoPP	*	0.0417
			DMSO vs. CoPP	***	0.0003
4B	MPP	one-way Anova with Bonferroni's multiple comparisons test	DMSO vs. CoPP	**	0.0017
4B	HPC-1	one-way Anova with Bonferroni's multiple comparisons test	G-CSF vs. CoPP	**	0.0075
			DMSO vs. CoPP	****	< 0.0001
4B	HPC-2	one-way Anova with Bonferroni's multiple comparisons test	G-CSF vs. CoPP	**	0.0014
			DMSO vs. CoPP	****	< 0.0001
4C	HPC-1	one-way Anova with Bonferroni's multiple comparisons test	DMSO vs. CoPP	**	0.0038
4C	HPC-2	one-way Anova with Bonferroni's multiple comparisons test	DMSO vs. CoPP	**	0.0073
4D	KLS-	one-way Anova with Bonferroni's multiple comparisons test	G-CSF vs. CoPP	**	0.0016
			DMSO vs. CoPP	****	< 0.0001
4D	EP	one-way Anova with Bonferroni's multiple comparisons test	NaCl vs. G-CSF	*	0.012
			DMSO vs. CoPP	*	0.0353
4D	MEP	one-way Anova with Bonferroni's multiple comparisons test	G-CSF vs. CoPP	****	< 0.0001
			DMSO vs. CoPP	****	< 0.0001
4D	GMP	one-way Anova with Bonferroni's multiple comparisons test	G-CSF vs. CoPP	*	0.0245
			DMSO vs. CoPP	***	0.0008
4E	KLS-	one-way Anova with Bonferroni's multiple comparisons test	NaCl vs. G-CSF	****	< 0.0001
			DMSO vs. CoPP		0.0729
			G-CSF vs. CoPP	**	0.0012
4E	EP	one-way Anova with Bonferroni's multiple comparisons test	NaCl vs. G-CSF	*	0.0176
			DMSO vs. CoPP	*	0.018
4E	MEP	one-way Anova with Bonferroni's multiple comparisons test	NaCl vs. G-CSF	***	0.0004
			DMSO vs. CoPP	**	0.0045
4E	GMP	one-way Anova with Bonferroni's multiple comparisons test	NaCl vs. G-CSF	***	0.0001
			G-CSF vs. CoPP	***	0.0001
5Ai	Granulocytes	one-way Anova with Bonferroni's multiple comparisons test	NaCl vs. G-CSF	*	0.0392
			NaCl vs. CoPP	****	< 0.0001
			G-CSF vs. CoPP	*	0.012
5AiI	KLS	one-way Anova with Bonferroni's multiple comparisons test	NaCl vs. CoPP	****	< 0.0001
			G-CSF vs. CoPP	****	< 0.0001
5B	2 weeks: WBC	one-way Anova with Bonferroni's multiple comparisons test	NaCl vs. CoPP	*	0.0022
5B	2 weeks: PLT	one-way Anova with Bonferroni's multiple comparisons test	NaCl vs. CoPP	**	0.0053
5B	2 weeks: HCT	one-way Anova with Bonferroni's multiple comparisons test	NaCl vs. CoPP	**	0.0031
5C	CD45+ cells	two-way Anova with Bonferroni's multiple comparisons test	NaCl vs. G-CSF	****	< 0.0001
			NaCl vs. CoPP	****	< 0.0001
			G-CSF vs. CoPP	**	0.0099

Appendix Table S2. Exact p values and statistical tests used – c.d.

Fig	Graph	Test	Comparison	Symbol	Exact p-value
5C	Granulocytes	two-way Anova with Bonferroni's multiple comparisons test	NaCl vs. G-CSF	***	0.0005
			NaCl vs. CoPP	****	< 0.0001
			G-CSF vs. CoPP	*	0.0271
5C	T cells	two-way Anova with Bonferroni's multiple comparisons test	NaCl vs. G-CSF	**	0.0093
			NaCl vs. CoPP	**	0.0028
5C	B cells	two-way Anova with Bonferroni's multiple comparisons test	NaCl vs. G-CSF	***	0.0008
			NaCl vs. CoPP	****	< 0.0001
			G-CSF vs. CoPP	*	0.0126
5D	KLS	Kruskal-Wallis with Dunn's multiple comparisons test	NaCl vs. G-CSF	*	0.0465
			NaCl vs. CoPP	*	0.011
5E	Mice with > 1% of GFP chimerism in PB	Fisher's exact test, two-tailed	G-CSF vs. CoPP		0.24
5F	CD45+ cells	Kruskal-Wallis with Dunn's multiple comparisons test	NaCl vs. CoPP	**	0.0033
5F	B cells	Kruskal-Wallis with Dunn's multiple comparisons test	NaCl vs. CoPP	**	0.0087
5I	KLS	Kruskal-Wallis with Dunn's multiple comparisons test	NaCl vs. CoPP	**	0.0074
6A	G-CSF	one-way Anova with Bonferroni's multiple comparisons test	DMSO vs. CoPP + IgG ctrl	**	0.001
			CoPP + IgG ctrl vs. CoPP + α-G-CSF	****	< 0.0001
6B	CD45+ cells	one-way Anova with Bonferroni's multiple comparisons test	DMSO vs. CoPP + IgG ctrl	****	< 0.0001
			CoPP + IgG ctrl vs. CoPP + α-G-CSF	****	< 0.0001
6B	Granulocytes	one-way Anova with Bonferroni's multiple comparisons test	DMSO vs. CoPP + IgG ctrl	***	0.0002
			CoPP + IgG ctrl vs. CoPP + α-G-CSF	****	< 0.0001
6B	HSC	one-way Anova with Bonferroni's multiple comparisons test	DMSO vs. CoPP + IgG ctrl	***	0.0003
			CoPP + IgG ctrl vs. CoPP + α-G-CSF	***	0.0006
6C	WBC	one-way Anova with Bonferroni's multiple comparisons test	HO-1-/-: DMSO vs. CoPP	**	0.0064
6D	%GRA	one-way Anova with Bonferroni's multiple comparisons test	HO-1+/+: DMSO vs. CoPP	**	0.0072
			HO-1-/-: DMSO vs. CoPP	*	0.0376
6E	G-CSF	one-way Anova with Bonferroni's multiple comparisons test	HO-1+/+: DMSO vs. CoPP	*	0.0248
6E	IL-6	one-way Anova with Bonferroni's multiple comparisons test	HO-1-/-: DMSO vs. CoPP		0.0558
6E	MCP-1	one-way Anova with Bonferroni's multiple comparisons test	HO-1+/+: DMSO vs. CoPP	**	0.0018
			HO-1-/-: DMSO vs. CoPP	*	0.0188
6F	CD45+ cells	two-way Anova with Bonferroni's multiple comparisons test	Nrf2+/+: DMSO vs. CoPP	*	0.0402
			Nrf2-/-: DMSO vs. CoPP	**	0.0017
6G	%GRA	two-way Anova with Bonferroni's multiple comparisons test	Nrf2+/+: DMSO vs. CoPP	****	< 0.0001
			Nrf2-/-: DMSO vs. CoPP	****	< 0.0001
6H	G-CSF	two-way Anova with Bonferroni's multiple comparisons test	Nrf2-/-: DMSO vs. CoPP		0.0674
6H	IL-6	two-way Anova with Bonferroni's multiple comparisons test	Nrf2+/+: DMSO vs. CoPP	*	0.049
			Nrf2-/-: DMSO vs. CoPP		0.0738
6H	MCP-1	two-way Anova with Bonferroni's multiple comparisons test	Nrf2-/-: DMSO vs. CoPP	****	< 0.0001

Appendix Table S2. Exact p values and statistical tests used – c.d.

Fig	Graph	Test	Comparison	Symbol	Exact p-value
EV3A	Granulocytes	one-way Anova with Bonferroni's multiple comparisons test	NaCl vs. CoPP	***	0.0002
			G-CSF vs. CoPP	*	0.029
EV3A	Ly6GmidSSCmid granulocytes	one-way Anova with Bonferroni's multiple comparisons test	NaCl vs. CoPP	*	0.0234
EV3A	Ly6GhiSSChi granulocytes	one-way Anova with Bonferroni's multiple comparisons test	NaCl vs. CoPP	****	< 0.0001
			G-CSF vs. CoPP	****	< 0.0001
EV3B	G-CSF	one-way Anova with Bonferroni's multiple comparisons test	NaCl vs. CoPP	*	0.0105
			G-CSF vs. CoPP	*	0.0203
EV3B	IL-6	one-way Anova with Bonferroni's multiple comparisons test	NaCl vs. CoPP	**	0.0035
			G-CSF vs. CoPP	**	0.0022
			G-CSF+IL-6 vs. CoPP	**	0.0022
EV3B	CCL2 (MCP-1)	one-way Anova with Bonferroni's multiple comparisons test	NaCl vs. CoPP	*	0.0382
			G-CSF vs. CoPP	*	0.0321
			G-CSF+IL-6 vs. CoPP	*	0.0359
S2	Immature granulocytes	two-way Anova with Bonferroni's multiple comparisons test	E.coli: NaCl vs. G-CSF	****	< 0.0001
			E.coli: G-CSF vs. CoPP	**	0.0024
			PMA: NaCl vs. G-CSF	***	0.0007
			PMA: DMSO vs. CoPP	***	0.0006
S2	Mature granulocytes	two-way Anova with Bonferroni's multiple comparisons test	PMA: NaCl vs. G-CSF	*	0.0124
			PMA: DMSO vs. CoPP		0.0742
S3A	KLS PB	one-way Anova with Bonferroni's multiple comparisons test	G-CSF vs. CoPP	****	< 0.0001
			DMSO vs. CoPP	**	0.0044
S3A	KLS BM	one-way Anova with Bonferroni's multiple comparisons test	DMSO vs. CoPP	*	0.0183
S3B	KLS CD34-	one-way Anova with Bonferroni's multiple comparisons test	NaCl vs. G-CSF	**	0.0092
			DMSO vs. CoPP	**	0.0044
S3B	KLS CD48-CD150+CD34-	one-way Anova with Bonferroni's multiple comparisons test	NaCl vs. G-CSF	*	0.0216
			DMSO vs. CoPP	**	0.0017
S5B	G-CSF	unpaired two-tailed T-test	Spleen: NaCl vs. CoPP		0.0568
S6	MON%	one-way Anova with Bonferroni's multiple comparisons test	Nrf2+/+ vs. Nrf2-/-	***	0.0003
S8B	ALP	one-way Anova with Bonferroni's multiple comparisons test	NaCl vs. G-CSF	**	0.0012
			DMSO vs. CoPP	**	0.0023
S8B	ALT	one-way Anova with Bonferroni's multiple comparisons test	G-CSF vs. G-CSF	***	0.0003
			DMSO vs. CoPP	***	0.0003
S8B	AST	one-way Anova with Bonferroni's multiple comparisons test	G-CSF vs. G-CSF	****	< 0.0001
			DMSO vs. CoPP	****	< 0.0001
S8B	BUN	one-way Anova with Bonferroni's multiple comparisons test	G-CSF vs. G-CSF	***	0.0005
S8B	CPK	one-way Anova with Bonferroni's multiple comparisons test	DMSO vs. CoPP	**	0.0059
S8B	GLU	one-way Anova with Bonferroni's multiple comparisons test	G-CSF vs. G-CSF	*	0.0178
S8B	LDH	one-way Anova with Bonferroni's multiple comparisons test	DMSO vs. CoPP	**	0.0018

Appendix Table S2. Exact p values and statistical tests used – c.d.
