# Supplementary material for: Cobalt protoporphyrin IX increases endogenous G‐CSF and mobilizes HSC and granulocytes to the blood
Source: EMBO Mol Med. 2019 Nov 11;11(12):e09571. doi: 10.15252/emmm.201809571 (PMC6895613; doi:10.15252/emmm.201809571)
Supplement: Supplementary file 2 — Expanded View Figures PDF [file EMMM-11-e09571-s002.pdf]

Expanded View Figures

Figure EV1. Schemes of mobilization experiments.

A CoPP and SnPP treatments in C3H mice; 2-month-old female C3H mice were treated with porphyrins or solvent control (DMSO) at days 1, 3, and 5. Mice were sacrificed 24 h after the last injection, and spleen, bone marrow, and peripheral blood were collected for further analysis.

B CoPP and G-CSF treatments in C57BL6xFVB and C57BL6 mice; 3- to 4-month-old C57BL6xFVB female mice were treated with NaCl, G-CSF, DMSO, or CoPP. Two- to 3-month-old female C57BL6 mice were treated with NaCl, G-CSF, G-CSF + IL-6, or CoPP. Mice were injected i.p. every day for 5 days and sacrificed 6 h after last injection.

C Neutralizing antibodies experiment; 2.5- to 3.5-month-old male C57BL6 mice were treated with anti-G-CSF, anti-G-CSF + anti-IL-6, or the control antibodies 1 h before CoPP treatment every day for 5 days, and sacrificed 6 h after last injection.

D CoPP treatment in HO-$1^{+/+}$ and HO-$1^{-/-}$ mice; 4- to 5-month-old male and female HO-$1^{+/+}$ and HO-$1^{-/-}$mice were injected with CoPP or DMSO every second day for 5 days, and sacrificed 24 h after last injection;

E CoPP treatment in $Nrf2^{+/+}$ and $Nrf2^{-/-}$ mice; 2-month-old female C57BL6 $Nrf2^{+/+}$ and $Nrf2^{-/-}$ mice were injected with CoPP or DMSO every day for 5 days, and sacrificed 6 h after last injection.

 © 2019 The Authors

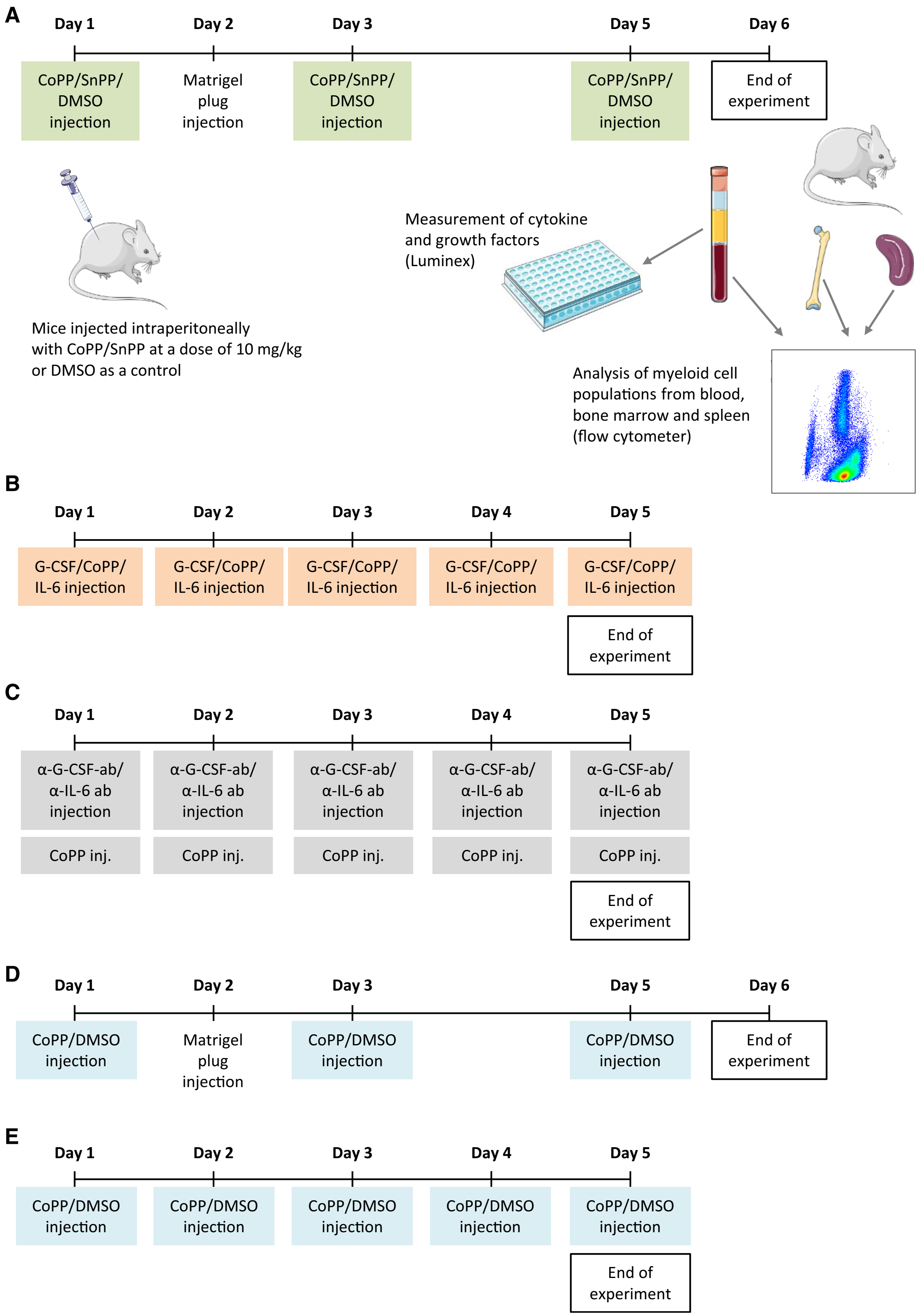

Figure EV1.

© 2019 The Authors

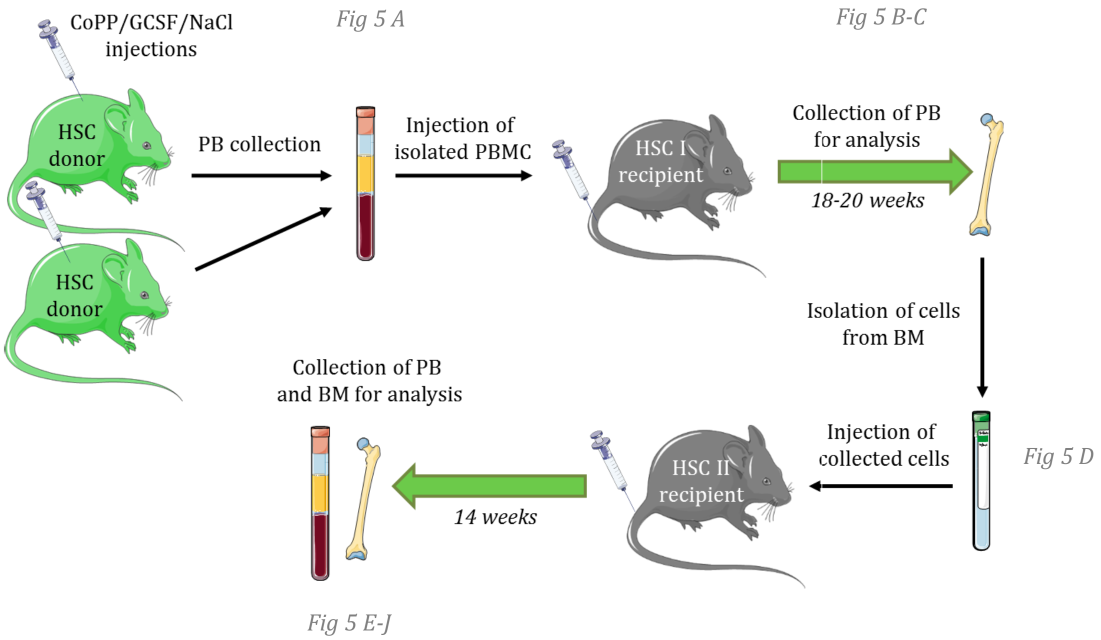

Figure EV2. Scheme of mobilized blood transplantation experiment.

PBMC from GFP+ mice mobilized with NaCl, G-CSF, or CoPP were isolated by density gradient centrifugation. 5×10^6 PBMC were transplanted to lethally irradiated GFP− recipients together with 1×10^5 BM supporting cells. Two independent experiments of primary transplantation were performed, and the results were pooled (NaCl: $n = 8$, G-CSF: $n = 7$, CoPP: $n = 9$); after 18–20 weeks, 10×10^6 of BM cells from primary recipients were transplanted to lethally irradiated secondary recipients.

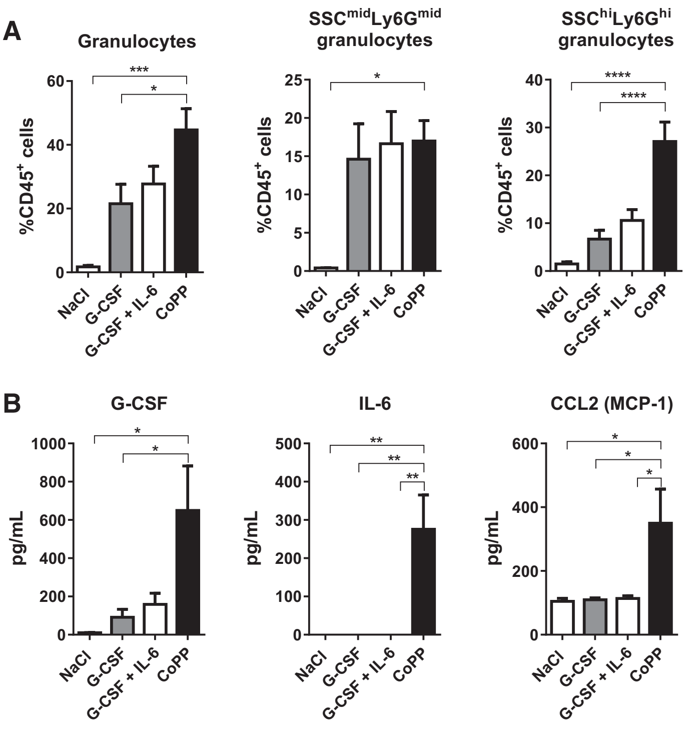

Figure EV3. Mobilization with G-CSF and IL-6 does not mimic the effect of CoPP.

C57BL6 mice were injected with G-CSF, G-CSF + IL-6, CoPP, or solvent control (NaCl) daily for 5 days. Samples were collected 6 h after the last injection.

A Mice treated with CoPP have higher percentage of total granulocytes and SSC^{hi}Ly6G^{hi} mature granulocytes, than mice treated with G-CSF only. Addition of recombinant IL-6 to G-CSF does not improve mature granulocytes mobilization.

B Plasma concentrations of G-CSF, IL-6, and MCP-1.

Data information: Results are shown as mean + SEM, one-way ANOVA with Bonferroni post-test, NaCl $n = 5$, G-CSF, G-CSF + IL-6 and CoPP: $n = 6$ mice per group. *$P \le 0.05$; **$P \le 0.01$; ***$P \le 0.001$; ****$P \le 0.0001$.

© 2019 The Authors
